# Human Metapneumovirus Glycoprotein G Disrupts Mitochondrial Signaling in Airway Epithelial Cells

**DOI:** 10.1371/journal.pone.0062568

**Published:** 2013-04-23

**Authors:** Xiaoyong Bao, Deepthi Kolli, Junping Ren, Tianshuang Liu, Roberto P. Garofalo, Antonella Casola

**Affiliations:** 1 Department of Pediatrics, The University of Texas Medical Branch at Galveston, Galveston, Texas, United States of America; 2 Department of Microbiology and Immunology, The University of Texas Medical Branch at Galveston, Galveston, Texas, United States of America; 3 Sealy Center for Vaccine Development, The University of Texas Medical Branch at Galveston, Galveston, Texas, United States of America; McMaster University, Canada

## Abstract

Human metapneumovirus (hMPV) is a recently identified RNA virus belonging to the *Paramyxoviridae* family. It is a common cause of respiratory tract infections in children, adults, and immunocompromised patients, for which no specific treatment or vaccine is available. Recent investigations in our lab identified hMPV glycoprotein G as an important virulence factor, as a recombinant virus lacking the G protein (rhMPV-ΔG) exhibited enhanced production of important immune and antiviral mediators, such as cytokines, chemokines and type I interferon (IFN) in airway epithelial cells, and expression of G protein alone inhibits cellular signaling dependent on retinoic induced gene (RIG)-I, a RNA helicase with a fundamental role in initiating hMPV-induced cellular responses. In this study, we have further investigated the mechanism underlying the inhibitory role of hMPV G protein on RIG-I-dependent signaling. We found that the interaction of hMPV G with RIG-I occurs primarily through the CARD domains of RIG-I N-terminus, preventing RIG-I association with the adaptor protein MAVS (mitochondrial antiviral signaling protein), recruitment of RIG-I to mitochondria, as well as the interaction between mitochondria and mitochondria-associated membrane (MAM) component of the endoplasmic reticulum (ER), which contains STINGS, an important part of the viral-induced RIG-I/MAVS signaling pathway, leading in the end to the inhibition of cytokine, chemokine and type I IFN expression. Mutagenesis analysis showed that hMPV G protein cytoplasmic domain played a major role in the observed inhibitory activity, and recombinant viruses expressing a G protein with amino acid substitution in position 2 and 3 recapitulated most of the phenotype observed with rhMPV-ΔG mutant upon infection of airway epithelial cells.

## Introduction

Human metapneumovirus (hMPV) was isolated for the first time in 2001 in the Netherlands, and has been quickly recognized as a leading cause of respiratory tract infections in infants, elderly and immunocompromised patients worldwide [1]. It is a negative single-stranded RNA virus of the family *Paramyxoviridae* and is closely related to the avian metapneumovirus (AMPV) subgroup C [2]. Among the hMPV proteins, the attachment glycoprotein G has been show to be critical for hMPV replication *in vivo*, as a recombinant hMPV in which the G protein was deleted (rhMPV-ΔG) exhibited reduced replication in the upper and lower respiratory tract of Syrian hamsters and African green monkeys [3], [4], therefore rhMPV-ΔG has been identified as a potential vaccine candidate. As hMPV G protein does not seem to play a major role in cellular attachment [4], [5], other mechanism(s) may be responsible for the observed attenuation of rhMPV-ΔG *in vivo*.

Toll-like receptor (TLR)- and RNA helicase-mediated signaling plays a critical role in host innate immunity against viral infection [6], [7]. The RNA helicases retinoic acid-inducible gene-I protein (RIG-I) and melanoma differentiation-associated protein 5 (MDA-5) are characterized by a conserved DExD/H box helicase domain, that binds different RNA moieties, a C-terminal regulatory domain (RD), and two in tandem caspase recruitment domains (CARDs) at the N-terminus [8]–[10]. RIG-I and MDA-5 initiate cellular signaling via binding of their helicase domain to viral RNA [11], [12], while their CARD domains mediates the interaction with the mitochondrial adaptor molecule MAVS (mitochondrial antiviral signaling protein), which is necessary for subsequent activation of downstream transcription factors such as Interferon Regulatory Factors (IRFs) and Nuclear Factor (NF)-κB [13], [14]. Mitochondria interacts with specific regions of the endoplasmic reticulum (ER), known as mitochondria-associated membranes (MAMs) [15], which contains STING (stimulator of interferon genes), an adaptor molecule that has been shown to interact with both RIG-I and MAVS, facilitating RNA- and DNA-induced cellular signaling [16], [17].

We have recently demonstrated that the hMPV G protein is an important virulence factor, as it inhibits host innate immune responses [18], [19]. Upon infection of airway epithelial cells, rhMPV-ΔG induces higher levels of cytokine, chemokine and type I interferon (IFN) secretion, and hMPV G protein expression results in inhibition of cellular signaling dependent on RIG-I [18], which play a fundamental role in initiating hMPV-induced cellular responses [20]. In this study, we have further investigated the mechanism underlying the inhibitory role of hMPV G protein on RIG-I signaling. We found that the interaction of hMPV G with RIG-I occurs primarily through the N-terminus domain, preventing RIG-I association with the adaptor protein MAVS, recruitment of RIG-I to mitochondria and formation of the mitochondrial signalosome, which include MAM, a fraction of the ER that contains STINGS, an important part of the viral-induced RIG-I/MAVS signaling pathway, consequently leading to the inhibition of cytokine, chemokine and type I IFN expression. Mutagenesis analysis showed that hMPV G protein cytoplasmic domain played a major role in the observed inhibitory activity, and recombinant viruses expressing a G protein with amino acid substitution in position 2 and 3 recapitulated most of the phenotype observed with rhMPV-ΔG upon infection of airway epithelial cells.

## Materials and Methods

### Cell lines and antibodies

LLC-MK2, A549 and 293 cells (all from ATCC, Manassas, VA) and BSR T7/5 cells (baby hamster kidney cells that constitutively express the T7 RNA polymerase) were maintained as described [18]. U4A cells, a JAK-1-deficient fibrosarcoma cell line (kindly provided by Dr. George Stark, Cleveland Clinic Foundation, Cleveland, OH), were cultured as previously described [21]. Monoclonal antibodies against lamin b and Flag were obtained from Sigma-Aldrich (Sigma, St. Louis, MO). Antibody against V5 was obtained from Invitrogen (Invitrogen, Carlsbad, CA). Antibodies against IRF-3, P50 and P65 were from Cell Signaling (Cell Signaling, Danvers, MA). Horseradish-coupled secondary antibodies were purchased from Santa Cruz (Santa Cruz, Santa Cruz, CA). The polyclonal rabbit anti-hMPV antibody was raised against sucrose-purified hMPVCAN-83. STING antibody was a generous gift of Dr. Barber, University of Miami, School of Medicine, Miami, Fl.

### Viral preparation and infection

Recombinant viruses derived from hMPVCAN-83 were propagated in LLC-MK2 cells at 35°C in the absence of serum and in the presence of 1 µg/ml of trypsin (Worthington, Lakewood, NJ), and were sucrose purified, as previously described [18], [22]. Viral titer was determined by immunostaining in LLC-MK2 cells, as previously described [18], [22]. To characterize rhMPV-G site mutant growth pattern, LLC-MK2 or Vero cell monolayers in 6-well plate were infected with rhMPV, wild type (WT) or mutants, at multiplicity of infection (MOI) of 0.01. An equivalent amount of sucrose solution was added to uninfected LLC-MK2 or Vero cells, as control (mock infection). After initial absorption, viral inoculum was removed and cells were supplied with fresh serum-free medium with trypsin. Viruses were harvested at different days post-infection (p.i.), and viral titer was determined by immunostaining as described [18], [22].

To investigate the role of rhMPV-G site mutants in modulating cellular signaling, A549 or U4A cell monolayers were infected with rhMPV, WT or G mutants, at a MOI of 2. Uninfected cells in serum-free media supplemented with trypsin, containing similar amount of sucrose as infected cells, were defined as mock-infected and used as a negative control.

### Plasmid construction

The C-terminus of hMPV G protein was cloned using the full length G in pCAGGS expression plasmid as template [18]. PCR was carried out using Pfu DNA polymerase (Stratagene, La Jolla, CA) following manufacturer's instruction. The primer sequences for V5-tagged G were: forward: 5′- ACGC*gaattc*ATGACAATACAAAAAACCTCATC -3′, and reverse: 5′- T*ctcgag*TCACGTAGAATCGAGACCGAGGAGAGGGTTAGGGATAGGCTTACCGTTTTGCATTGTGCTTACAGATGCCTG-3′. Italicized letters indicate restriction enzyme site. Underlined letters indicate V5 sequence. The cloned V5-tagged C-termini was first cloned into the TOPO cloning vector, and then cut by EcoRI and XhoI and subcloned into pCAGGS vector.

Site mutants of G protein N-terminus were generated by replacing Glu 2, Val 3, Lys 4, Val 5, Glu 6, Asp 7, IIe 8 or Arg 9 with Ala using a multiple-site mutagenesis kit (Agilent, La Jolla, CA) and the full-length G in pCAGGS vector as template. All plasmid constructs were verified by sequencing performed by the protein chemistry core laboratory at UTMB. Information regarding the primers used to generate these mutants is available upon request.

### Recovery of mutant rhMPV

Construction of G mutant cDNA was performed using a similar approach we used to generate rhMPV-ΔG [18]. Previously, we inserted the complete hMPV genome into pBSKSII vector by sequential ligation of three fragments amplified from a cDNA template. The G protein gene is contained within fragment II, together with hMPV F, M2 and SH genes. To generate a rhMPV containing Ala residues instead of Glu 2 or Val 3 residues, we used the same approach described above, using fragment II in TOPO vector as template. The primer used to mutate Glu 2 to Ala in G is 5′-GGGACAAGTAGTTATG*G* ***CT***GT**C**AAAGTAGAGAACATTCGAGCAATAGAC-3′. The primer to change Val 3 to Ala is 5′- GGGACAAGTAGTTATGGAG*G* ***C*** *G*AAAGTAGAGAACATTCGAGCAATAGAC-3′. Italicized letters encode Ala. Bold letters indicate mutated residues either for Ala coding or silent mutation for cloning screening. The mutated fragment II was then cloned back into pBSKSII using NheI and KpnI sites as described [18]. Mutated recombinant viruses were recovered by transfection of BSR T7/5 cells with genomic plasmid together with plasmids encoding supportive proteins as previously described [18], [22]. Trypsin was added on day 3 post-transfection to a final concentration of 1 µg/ml, then cell-medium mixtures were passed onto fresh LLC-MK2 cells and incubated at 35°C. Typical viral CPE were usually observed around day 5 -6 post-infection (p.i.). Genomic mutations were confirmed by viral RNA sequencing. The recovered viruses were then amplified for two passages in LLC-MK2 cells and saved as stock viral preparations. Viruses with no more than 4–5 passages were used in all experiments.

### Reporter gene assays

To investigate the effect of deletions or site mutations of hMPV G protein on RIG-I dependent gene transcription, logarithmically growing A549 cells were co-transfected in triplicate in 24 well plates with the a plasmid containing the N-terminus of RIG-I (a generous gift of Michael Gale, University of Washington), pCAGGS containing full length, truncated or mutated G protein or the control vector, and a luciferase reporter plasmid containing the IFN-β promoter, using FuGene 6 (Roche Diagnostic Corp., Indianapolis, Ind.). Cells were harvested 30 h post-transfection to independently measure luciferase and β-galactosidase reporter activity, as previously described [23]. To investigate the role of hMPV G site mutants on NF-κB/IRF-dependent gene transcription, A549 were transfected as described above with a luciferase reporter gene plasmid containing multiple copies of NF-κB or IRF-3 binding site, as previously described [18], [22]. The next day, cells were infected with recombinant hMPV, WT or harboring G site mutation and harvested at 24 h p.i. to measure the luciferase activity, as described above. Uninfected plates served as control. All experiments were performed two to three times.

### Mitochondria isolation

Mitochondria were isolated using the Qproteome Mitochondria Isolation Kit from Qiagen (Qiagen, Valencia, CA), according to manufacturer's instructions. Purity of mitochondria was assessed by the absence of β-actin, a marker of cytoplasmic proteins. Isolated mitochondria were resuspended in SDS sample buffer for Western blot analysis.

### Co-immunoprecipitation assays

Logarithmically growing 293 cells in 6-well plate were cotransfected with pcDNA6 containing C-terminus or N-terminus Flag-tagged RIG-I domain (a generous gift of Michael Gale, University of Washington) and either pCAGGS encoding V5-tagged G protein or the empty vector, used as a negative control. Cells were harvested 30 h post-transfection and immunoprecipitation was carried out using immunoprecipitation Kit from Roche (Cat # 11719, Indianapolis, IN) and an antibody against either V5 or Flag or an isotype antibody control, as previously described [18], [24]. Immunocomplexes were subjected to SDS-PAGE and Western blot analysis.

To investigate the molecular mechanisms underlying the disruption of RIG-I-mediated signaling, logarithmically growing A549 cells in 6-well plates were co-transfected with a fixed amount of plasmids encoding Flag-tagged RIG-I and EGFP-tagged MAVS (a generous gift of Kui Li, University of Tennessee Health Science Center), or their control vectors, and increasing concentrations of V5-tagged G expression plasmid. Cells were harvested 30 h after transfection and immunoprecipitation was carried out using anti-Flag antibody to pull down RIG-I, followed by Western blot using anti-EGFP or anti-V5 antibody to detect associated MAVS or hMPV G protein, respectively.

### Western blot analysis

Nuclear extracts of uninfected and infected cells were prepared using hypotonic/nonionic detergent lysis, according to Schaffner protocol [25]. To prevent contamination with cytoplasmic proteins, isolated nuclei were purified by centrifugation through 1.7 M sucrose buffer A for 30 minutes, at 12,000 rpm, before nuclear protein extraction, as previously described [18], [25]. Total cell lysates of uninfected and infected cells were prepared by adding ice-cold lysis buffer (50 mM Tric-HCl, pH 7.4, 150 mM NaCl, 1 mM EGTA, 0.25% sodium deoxycholate, 1 mM Na_3_VO_4_, 1 mM NaF, 1% Triton X-100 and 1 μg/ml of aprotinin, leupeptin and pepstatin). After incubation on ice for 10 min, the lysates were collected and detergent insoluble materials were removed by centrifugation at 4°C at 14,000 g. After normalizing for protein content, using Bio-Rad, Hercules, CA. Nuclear extracts or total cell lysates were fractionated by SDS-PAGE, and transferred to polyvinylidene difluoride membranes. Membranes were blocked with 5% milk in TBS-Tween and incubated with the proper primary and secondary antibodies according to manufacturer's instruction.

### ELISAs

IFN-β, IL-8 and RANTES levels in cell supernatants were quantified by ELISA (IFN-β: PBL Biomedical Laboratories, Piscataway, NJ; IL-8 and RANTES: R&D, Minneapolis, MN).

### Statistical Analysis

Statistical significance was analyzed using analysis of variance (ANOVA). *P* value of less than 0.05 was considered significant. Mean ± standard error (SE) is shown.

## Results

### G protein inhibits the association of RIG-I with MAVS

The RIG-I/MAVS pathway plays an essential role in initiating cellular signals leading to the activation of transcription factors and subsequent induction of inflammatory/immune and antiviral mediators in response to viral infections [12], [26], [27]. In recent investigations, we found that this pathway is necessary for hMPV-induced gene expression in airway epithelial cells [20], and it is targeted by hMPV G protein during airway epithelial cell infection, as its expression blocks RIG-I-dependent gene transcription [18]. We have shown that hMPV G protein can bind RIG-I in an overexpression system and in the context of infection [18], however, the molecular mechanism underlying the G protein inhibitory activity on RIG-I-mediated signaling was not investigated.

**Figure 1:**
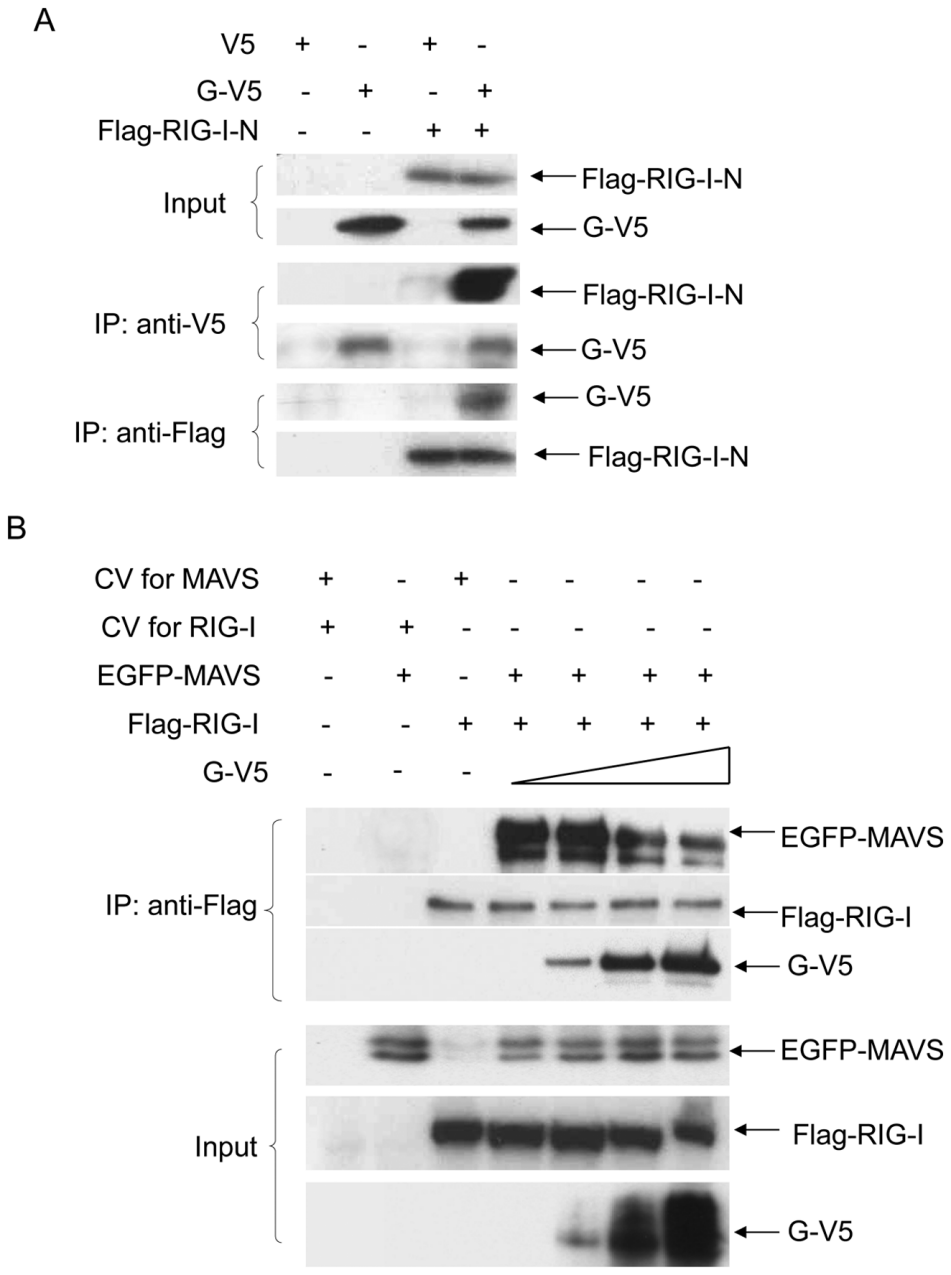
G protein blocks the interaction between RIG-I and MAVS. (**A**) 293 cells were transfected with plasmids encoding Flag-tagged RIG-I-N and V5-tagged G or their control vectors. Total cell lysates were immunoprecipitated with anti-V5 antibody followed by Western blot using anti-Flag antibody to detect RIG-I-N. Reverse immunoprecipitation was also done, where RIG-I-N was immunoprecipitated using anti-Flag antibody and G protein was then detected using anti-V5 antibody. Total cell lysates were subjected to Western blot to determine levels of hMPV G and RIG-I-N expression. (**B**) 293 cells were transfected with a fixed amount of plasmids encoding Flag-tagged RIG-I and EGFP-tagged MAVS, or their control vectors, and increasing concentrations of a plasmid expressing V5-tagged G. Total cell lysates were immunoprecipitated with anti-Flag antibody to pull down RIG-I, followed by Western blot using anti-EGFP or anti-V5 antibody to detect associated MAVS and G, respectively. Data are representative of two independent experiments.

To determine whether hMPV G protein could bind to RIG-I CARD domains, leading to the disruption of RIG-I/MAVS association, 293 cells were transfected with expression plasmids encoding V5-tagged G and Flag-tagged N-terminus of RIG-I (RIG-I-N, which contains the two CARD domains of RIG-I). Vectors expressing V5 or Flag only were used as negative controls. Cells were lysed followed by immunoprecipitation using anti-V5 antibody and immunoprecipitated complex was subjected to Western blot analysis using anti-Flag antibody. The results showed that hMPV G protein interacts with RIG-I-N (Fig. 1A), an interaction confirmed by reverse immunoprecipitation, using anti-Flag to precipitate expressed RIG-I-N and anti-V5 antibody to detect G protein by Western blot (Fig. 1A). When a similar experiment was performed using RIG-I C-terminus, only a very weak interaction was detected (data not shown). A similar experiment performed using a V5-tagged F expression plasmid and Flag-tagged RIG-I N did not show any interaction between the two proteins (data not shown and [18] ).

To investigate whether the interaction between hMPV G and RIG-I could prevent its association with MAVS, 293 cells were transfected with Flag-tagged RIG-I, EGFP-tagged MAVS, and increasing concentrations of V5-tagged G. Vectors expressing Flag or EGFP or V5 only were used as negative controls. G/RIG-I/MAVS complex was immunoprecipitated using anti-Flag antibody and the immunoprecipitated complex was subjected to Western blot analysis using anti-EGFP and anti-V5 antibodies. The results showed that RIG-I interacted with MAVS in the absence of G protein expression (Fig. 1B, lane 4). However, the abundance of MAVS in the IP complex progressively decreased as G protein expression levels increased (Fig 1C. lane 5–7), demonstrating that hMPV G protein disrupts RIG-I/MAVS association.

### G protein prevents the recruitment of RIG-I and ER-MAM to mitochondria in response to hMPV infection

**Figure 2:**
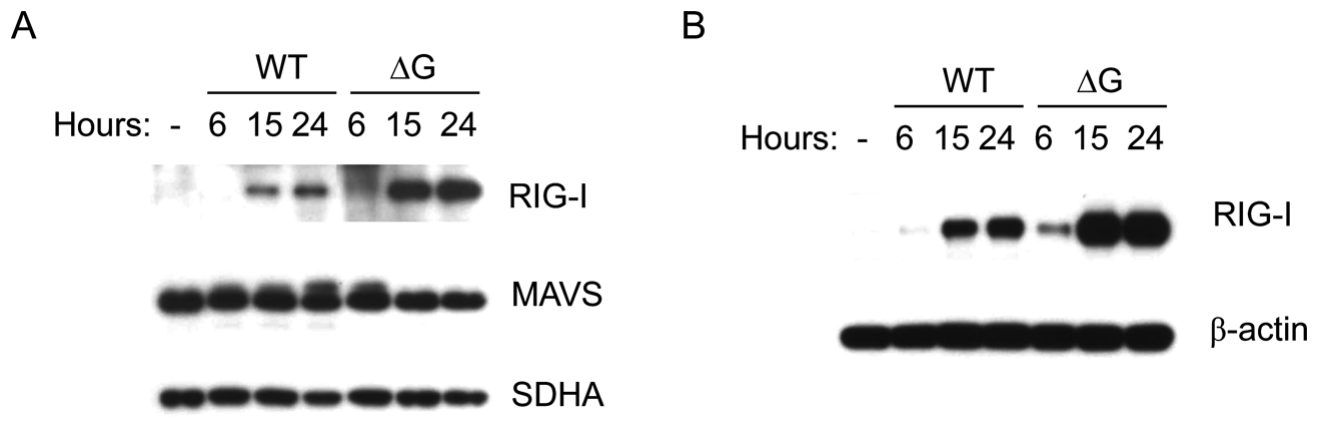
G protein prevents RIG-I recruitment to mitochondria in A549 cells. A549 cells were infected with rhMPV-WT or -ΔG, and harvested at different time p.i. to prepare total cell lysates or to purify mitochondria. Levels of RIG-I and MAVS in mitochondria (**A**) or RIG-I in cell lysates (**B**) were determined by Western blot. Membranes were stripped and reprobed with anti- SDHA or anti-β-actin antibody, respectively, as loading controls. Data are representative of two independent experiments.

To confirm that G protein affects RIG-I-MAVS interaction in airway epithelial cells, we investigated RIG-I recruitment to mitochondria in response to rhMPV-WT or -ΔG infection. Uninfected and infected A549 cells were harvested at various times p.i. to prepare mitochondrial fraction. We found that rhMPV-ΔG-infected cells had more RIG-I recruited to mitochondria than WT-infected cells at all times p.i. There was also a slight reduction in abundance of MAVS at 15 and 24 h p.i., with no change in levels of the mitochondrial protein SDHA (succinate dehydrogenase complex, subunit A), used as loading control (Fig. 2A), suggesting that hMPV G protein expression inhibits RIG-I/MAVS interaction in infected cells. However, total cell lysate analysis showed that rhMPV-ΔG infection induced significantly more RIG-I expression than the WT virus (Fig. 2B), likely through the greatly enhanced type I IFN production induced by rhMPV-ΔG [18], raising the question whether increased mitochondrial RIG-I levels only reflected increased cellular levels.

**Figure 3:**
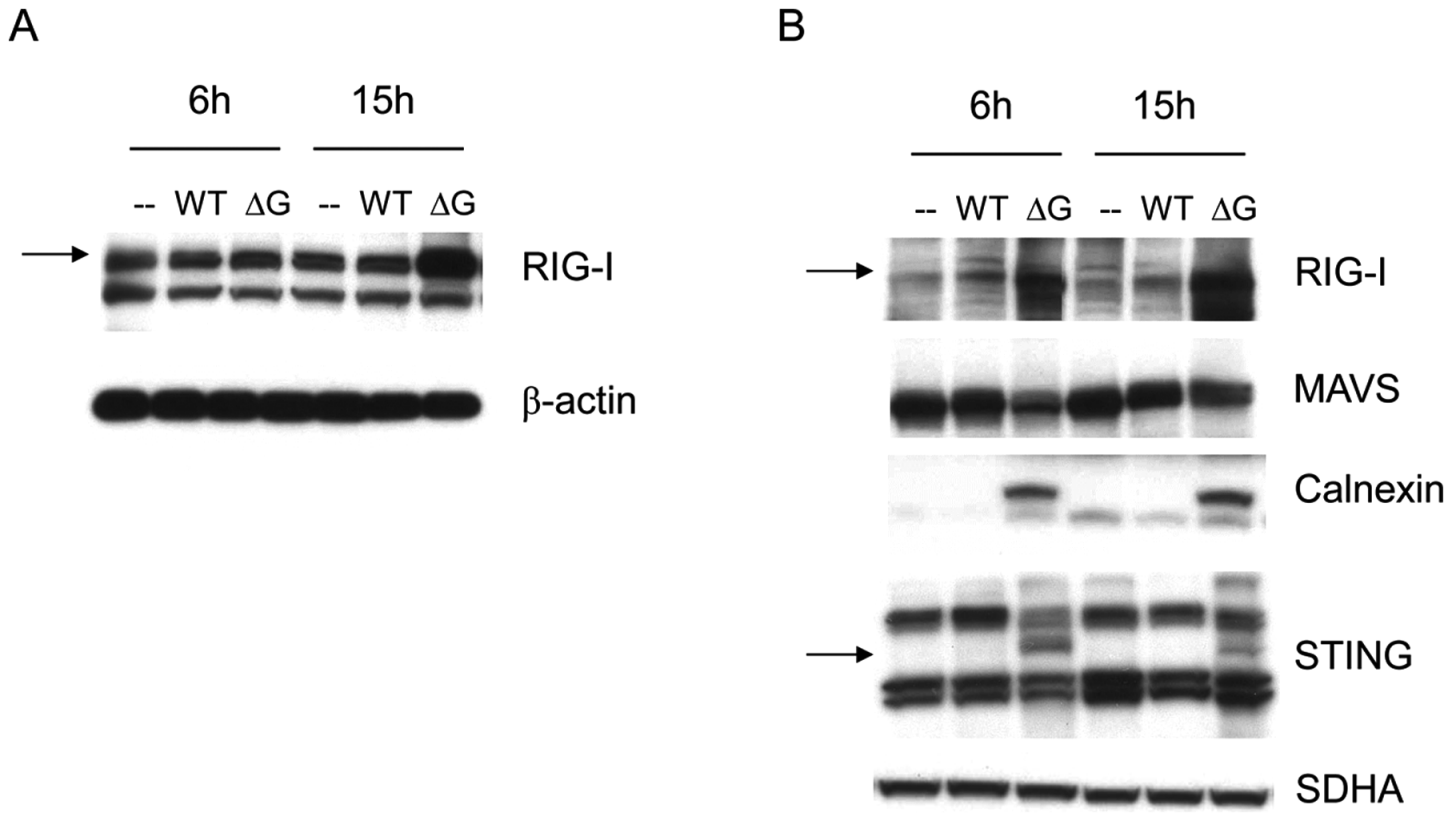
G protein prevents RIG-I and ER-MAM recruitment to mitochondria in U4A cells. U4A cells were infected with rhMPV-WT or -ΔG, and harvested at different time p.i. to either prepare total cell lysates or to purify mitochondria. Levels of RIG-I, MAVS, STING and calnexin levels in mitochondria (**A**) or RIG-I in cell lysates (**B**) were determined by Western blot. Membranes were stripped and reprobed with anti-beta actin or anti-SDHA antibody, respectively, as loading controls. Data are representative of two independent experiments.

To investigate the effect of hMPV G on RIG-I/MAVS interaction in the absence of IFN signaling, we used U4A cells, which lack JAK1 expression, a molecule necessary for type I, II and III IFN signaling [28]. U4A cells response to WT and ΔG infection was similar to the one we have previously observed in A549 cells [18], with ΔG-infected cells producing more IL-8 and RANTES than WT-infected U4A (supplementary Fig. 1A and B), suggesting that G protein modulates cellular responses in these cells too. As in A549 cells, viral protein expression was somewhat lower in rhMPV-ΔG infected cells compared to WT virus, so increased proinflammatory secretion was not due to increased viral replication (supplementary Fig. 1C and [18]). In absence of IFN signaling, there was no significant induction of RIG-I in both rhMPV-WT- and ΔG-infected cells at early time points of infection, although there was still a slight increase in RIG-I levels at 15 h p.i. in ΔG-infected cells (Fig. 3A), which was no longer observed at 24 h p.i. (data not shown). There was significantly more RIG-I recruited to mitochondria in rhMPV-ΔG-infected cells compared to WT-infected cells, at all time points of infection, indicating that G protein expression correlates with inhibition of RIG-I migration to mitochondria (Fig. 3B). As in A549 cells, mitochondrial MAVS protein levels were decreased in response to rhMPV-ΔG infection, compared to WT, likely reflecting enhanced degradation, which has been recently shown to be required to activate downstream signaling leading to type I IFN production [29].

To determine whether lack of RIG-I recruitment leads to disruption of viral-induced mitochondrial signalosome, we investigated ER-MAM recruitment to mitochondria. Calnexin is a marker ER-MAM, as well as STING, an adaptor molecule that interacts with RIG-I/MAVS and facilitates viral-induced signaling through this pathway [16], [30]. We found that calnexin and STING levels on mitochondria were also greatly reduced in the presence of hMPV G protein expression (Fig. 3B), supporting the hypothesis that G protein inhibits formation of viral-induced mitochondrial signaling platform.

### G protein N terminus plays a major role in inhibiting RIG-I-dependent signaling

**Figure 4:**
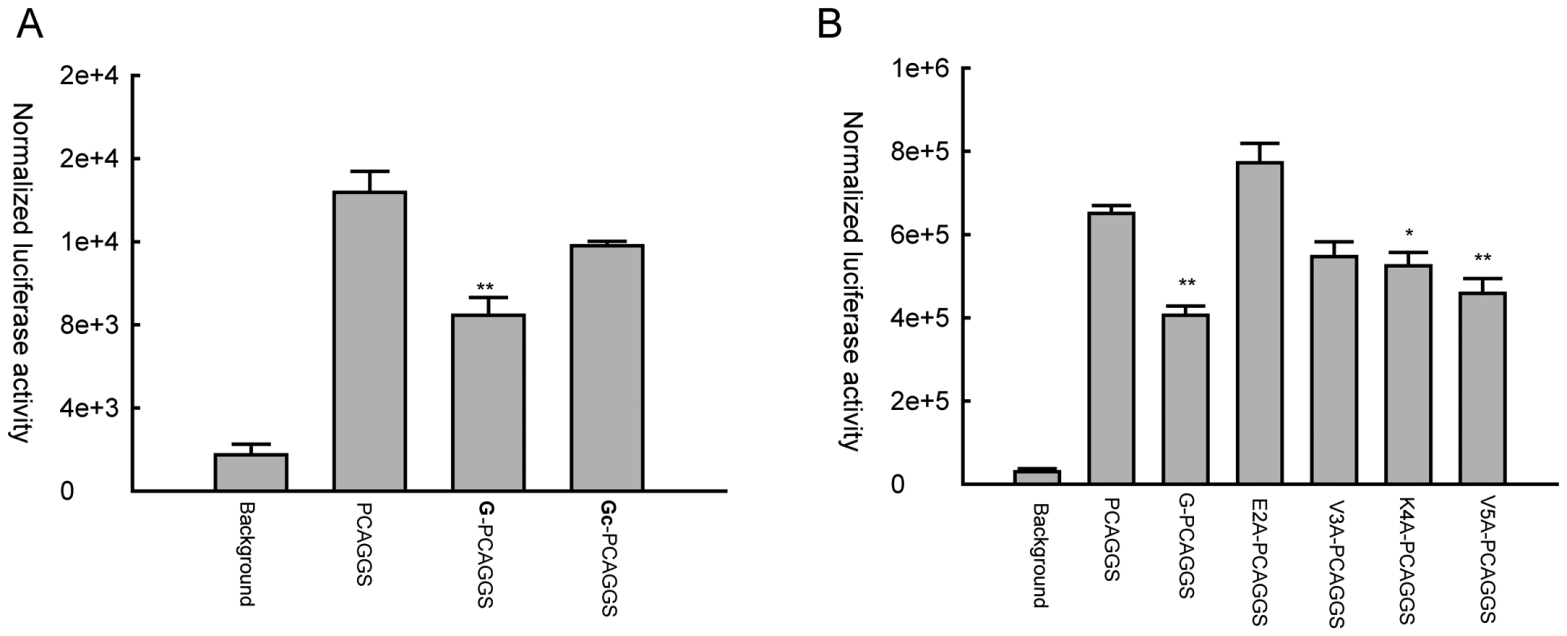
Amino acid residues Glu 2 and Val 3 of hMPV G cytoplasmic domain are responsible for the inhibition of RIG-I-dependent gene transcription. A549 cells were co-transfected with a luciferase reporter plasmid containing the human IFN-β promoter, a RIG-I expression plasmid and a plasmid containing either full length G or G extracellular domain (Gc) (A) or G mutants carrying Ala mutation of individual amino acids, as indicated (B), or the corresponding empty vector. Cells were harvested 30 h post-transfection to measure luciferase activity. For each plate luciferase was normalized to the β-galactosidase reporter activity. Data are representative of two independent experiments and are expressed as mean ± SE of normalized luciferase activity. **, *P*<0.01 and *, *P*<0.05, relative to pCAGGS+RIG-I group.

G protein is a type II mucin-like glycosylated transmembrane protein, with the N-terminus facing intracellular space and the C terminus oriented externally [31]. As RIG-I and MAVS are cytoplasmic proteins, we hypothesized that hMPV G inhibitory activity occurs through its intracellular domain. To test this hypothesis, we generated a deleted hMPV G mutant that lacks the intracellular and transmembrane domain (hMPV Gc) and tested its ability to inhibit RIG-I-dependent IFN-β gene transcription in reporter gene assays. A549 cells were transfected with the IFN-β gene promoter linked to the luciferase reporter gene, RIG-I and plasmids containing either the full length G protein or the Gc fragment, which was expressed at levels comparable to full length G (supplementary Fig. 2). The results showed that expression of hMPV Gc did not significantly inhibit RIG-I-induced IFN-β gene transcription, compared to full length G (Fig. 4A). We then generated hMPV G site mutants of amino acids 2 to 9 by alanine scanning mutagenesis, as previously described [32], and we performed the same experiment as above. We found that replacement of Glu 2 and Val 3 with Ala abolished or significantly reduced the inhibitory effect of hMPV G on RIG-I-induced IFN-β gene transcription, while the other mutants did retain the ability to inhibit RIG-I signaling (Fig. 4B and data not shown for Glu6A, Asp7A, IIe8A and Arg9A), suggesting that the amino acid residues in position two and three of the hMPV G cytoplasmic domain are critical for the inhibitory function of G protein on cellular signaling.

### Glu 2 and Val 3 G residues modulate hMPV-induced cellular signaling

**Figure 5:**
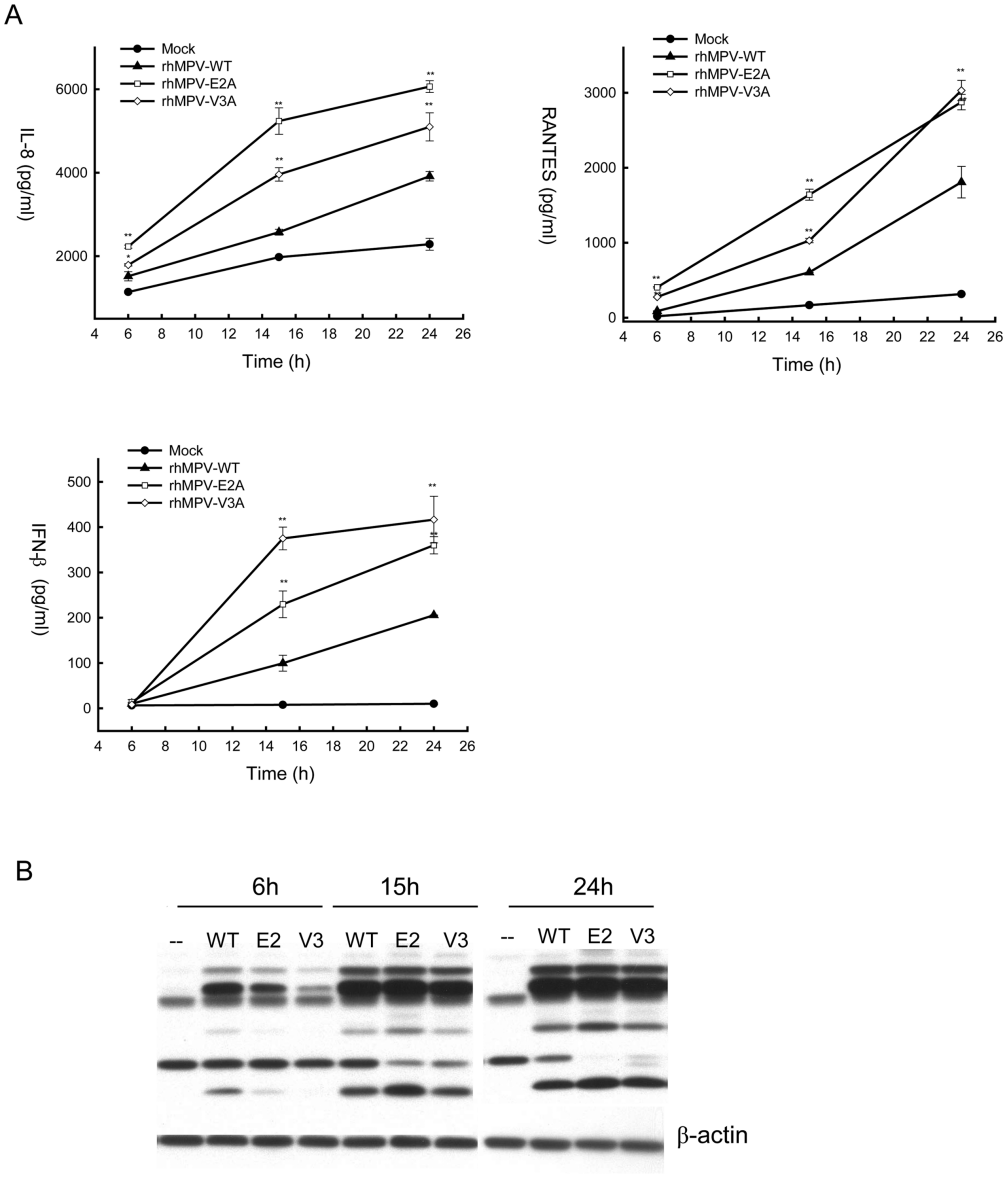
Effect of G protein Glu 2 and Val 3 residue mutations on cytokine and chemokine secretion. A549 cells were infected with rhMPV-WT or G mutant viruses and harvested at various times p.i. to collect cell supernatants and to prepare total cell lysates. IL-8, RANTES and IFN-β secretion was measured in cell supernatants by ELISA (**A**), while viral protein expression was determined by Western blot assay in cell lysates (**B**). Data are representative of two independent experiments. **, *P*<0.01 relative to rhMPV-WT.

To confirm the role of Glu 2 and Val 3 G residues in cellular signaling inhibition, we generated recombinant rhMPV harboring site mutations of G protein, where Glu 2 or Val 3 was replaced with Ala. We named these mutants rhMPV-E2A and rhMPV-V3A, respectively, and we tested the effect of these two mutations on hMPV-induced cellular signaling. A549 cells were infected with rhMPV-E2A, V3A or WT, and cell supernatants were harvested at various times p.i to measure IL-8, RANTES and IFN-β by ELISA. As shown in Fig. 5A, rhMPV-E2A and -V3A resulted in significantly higher levels of chemokine and type I IFN secretion at all time points of infection, compared to rhMPV-WT. This effect was not due to differences in viral replication between rhMPV-WT and the mutants, as viral protein expression during the course of infection was similar for all three viruses (Fig. 5B) and viral titers assessed in multicycle replication assays were also similar between rhMPV-WT and the G protein site mutants (data not shown).

**Figure 6:**
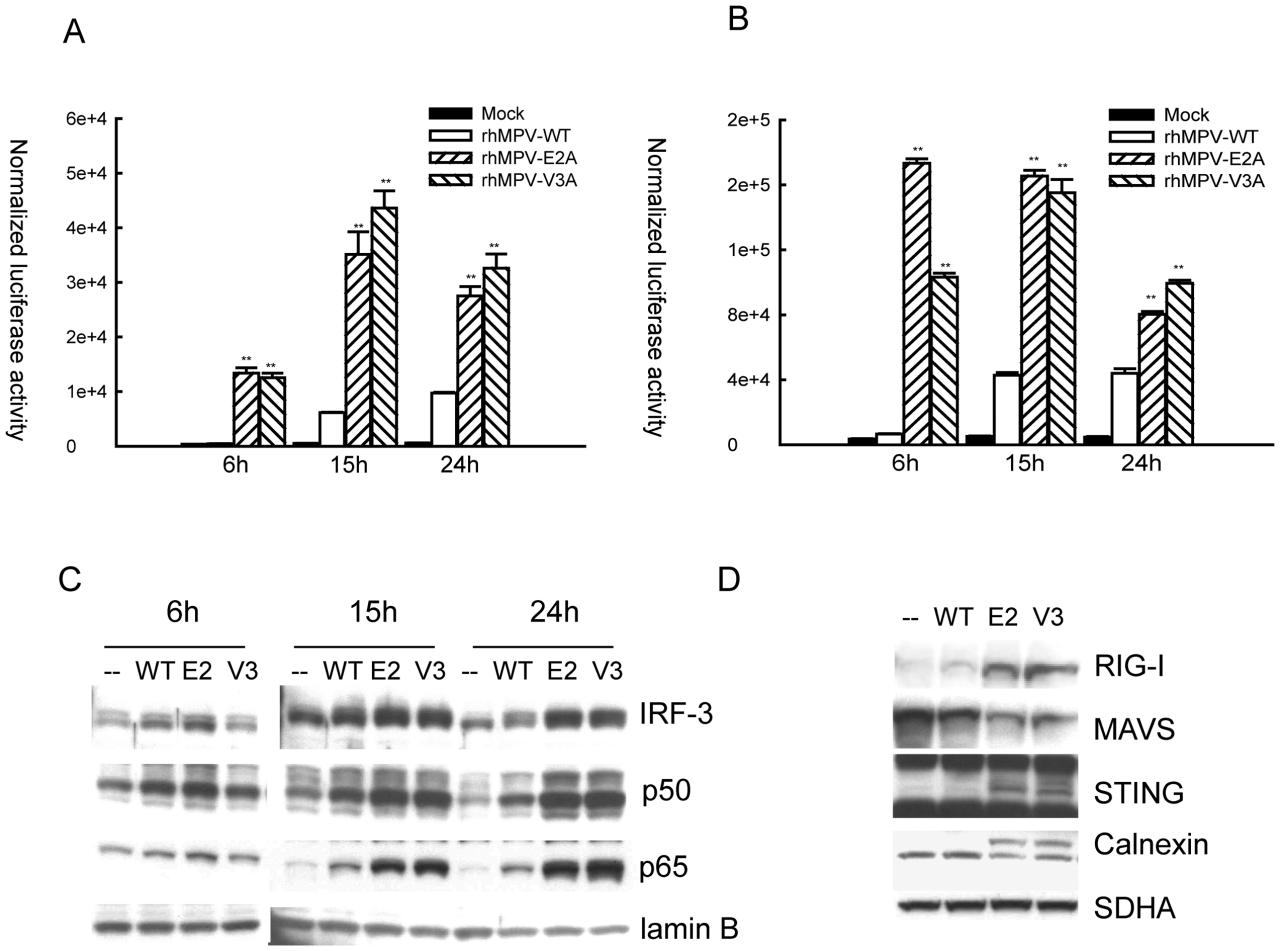
Glu 2 and Val 3 residues of G protein suppress IRF-3 and NF-κB activation by inhibiting mitochondrial signalosome formation. A549 cells were cotransfected with a luciferase reporter plasmid containing the RANTES ISRE site (**A**) or multimers of the IL-8 NF-κB site (**B**). At 24 h post-transfection, cells were infected with rhMPV-WT or rhMPV-E2Aor rhMPV-V3A, at MOI of 2, and harvested at different times p.i. to measure luciferase activity. Uninfected plates served as controls. For each plate luciferase was normalized to the β-galactosidase reporter activity. Data are representative of two independent experiments and are expressed as mean± SE. **, *P*<0.01, relative to rhMPV-WT-infected A549 cells. (**C**) A549 cells were infected with rhMPV-WT or rhMPV-E2A, or rhMPV-V3A, at MOI of 2, for various lengths of time and harvested to prepare nuclear extracts. Equal amounts of protein from uninfected and infected cells were analyzed by Western blot using an anti-IRF-3, anti-p50 or anti-p65 antibody. Membranes were stripped and reprobed for lamin b, as control for equal loading of the samples. Data shown are representative of two independent experiments. (**D**) U4A cells were infected with rhMPV, WT or G mutants and harvested at 6 h p.i. to purify mitochondria. The abundance of mitochondria-associated RIG-I, MAVS, STING and calnexin was investigated by Western blot. Membranes were stripped and reprobed with anti-SDHA, as control for comparable loading of samples. Data shown are representative of two independent experiments.

We and others have shown that transcription factors belonging the NF-κB and IRF families control pro-inflammatory and antiviral gene expression in response to paramyxoviruses [33]–[38]. To determine whether hMPV G site mutations affected transcription factor activation, we used reporter gene assays to investigate NF-κB- and IRF-dependent gene transcription, and Western blot assays to look at changes in their nuclear levels. A549 cells were transfected with a construct containing multiple copies of either the RANTES promoter ISRE site, which binds IRF proteins, or the IL-8 NF-κB site linked to the luciferase reporter gene, and infected with rhMPV-WT or G protein site mutants. Cells were harvested at the indicated times p.i. to measure luciferase activity. Both IRF- and NF-κB-driven luciferase activity (Fig. 6A and B, respectively) was significantly higher in A549 cells infected with rhMPV-E2A or -V3A compared to hMPV-WT-infected cells at all times p.i., confirming that E2 and V3 residues of hMPV G are responsible for the inhibitory activity of G protein on cytokine and chemokine gene transcription.

To investigate changes in IRF- and NF-κB activation, A549 cells were infected with rhMPV-WT or G site mutants and harvested at different times p.i. to prepare nuclear extracts. Nuclear abundance of IRF-3, p65 and p50 (the NF-κB family members activated by hMPV infection of airway epithelial cells [20], [38]) was then investigated by Western blot analysis. We found that there was a significant increase in nuclear translocation of all three transcription factors at all time points of rhMPV-E2A and V3A infection, compared to rhMPV-WT (Fig. 6C).

To confirm a role for Glu 2 and/or Val 3 residues of hMPV G in assembly of the mitochondrial signalosome, U4A were infected with rhMPV-WT, -E2A or -V3A and levels of RIG-I and ER-MAM recruited to purified mitochondria were analyzed by Western blot. We found that rhMPV-E2A- and -V3A- infected cells had significantly higher levels of RIG-I, STING and calnexin associated with mitochondria than WT-infected cells (Fig. 6D), and reduced MAVS levels, further supporting the concept that these two amino acid residues are critical for hMPV G protein inhibitory activity on cellular signaling.

Last, to investigate whether the Glu 2 and/or Val 3 residues of hMPV G was responsible for preventing RIG-I association with MAVS, 293 cells were transfected with Flag-tagged RIG-I, EGFP-tagged MAVS, V5-tagged G wild type and mutants. HMPV V5-tagged N protein was used as negative control. RIG-I/MAVS complex was immunoprecipitated using anti-Flag antibody and the immunoprecipitated complex was subjected to Western blot analysis using anti-EGFP and anti-Flag antibodies. As shown in supplementary Fig. 3, expression of wild type G, but not the E2 or V3 mutant, or the unrelated N protein, resulted in inhibition of RIG-I/MAVS interaction, confirming that these two aa residues play a key role in inhibiting RIG-I-dependent cellular signaling.

## Discussion

Upon infection, viruses are rapidly recognized by the innate immune system PRRs (Pattern recognition receptors), including TLRs and RIG-I-like receptors (RLRs), and activate a cascade of cellular responses, leading to antiviral and immune gene expression. Innate cellular signaling in response to viral infection is cell-type dependent and we have recently shown that, in the context of hMVP infection, TLR-4 and RIG-I play a major role in viral-induced signaling in immune cells and airway epithelial cells, respectively [19], [20]. Similar to many other viruses, hMPV has evolved strategies to efficiently inhibit antiviral responses. We have recently shown that hMPV strategically uses G protein to block these cell-specific signaling to efficiently evade host defenses. In dendritic cells, hMPV G protein inhibits LPS-dependent signaling, leading to enhanced cytokine and chemokine production [19], while in airway epithelial cells it affects RIG-I-dependent signaling, leading to a generalized inhibition of proinflammatory and immunomodulatory cellular responses [18]. In this study, we found that hMPV G protein expression results in the disruption of mitochondrial signaling platform formation. Following RIG-I engagement, MAVS at the mitochondrial surface serves as a recruitment platform for the assembly and activation of a signaling complex involving NEMO and TBK1, which is required for NF-κB and IRF-3 activation. Triggering of the RIG-I/MAVS pathway also results in recruitment to the mitochondria of a ER fraction known as ER-MAM, which contains a transmembrane protein, STING (also reported as MPYS, ERIS, TMEM173 or MITA), that has been show to play an important role in RLR signaling and is involved in RNA virus recognition, as STING-deficient MEFs showed reduced type I IFN response to vesicular stomatitis virus (VSV) and Sendai virus [16], [17]. Our study shows that hMPV G interacts with RIG-I, primarily through its N-terminus domain, preventing RIG-I association with the adaptor protein MAVS, recruitment of RIG-I and ER-MAM to mitochondria, ultimately leading to reduced activation of IRF-3 and NF-κB, as well as cytokine and chemokine expression, in response to hMPV infection. Recent findings have suggested that proteasomal degradation of MAVS is necessary for optimal transcription factor activation, possibly by allowing NEMO/TBK1 complex to translocate from the mitochondria into the cytosol [29]. This is consistent with our findings of reduced mitochondrial MAVS levels in rhMPV-ΔG infected cells, in which RIG-I and ER-MAM components were recruited to mitochondria, indicating fully functional mitochondrial signalling.

In order to dissect the functional domains of hMPV G protein, to better understand the mechanism leading to the inhibition of immune and antiviral gene expression, we performed *in vitro* mutagenesis, focusing on the N-terminal domain of G, as it faces the intracellular compartment, therefore being more likely to interact with the RIG-I/MAVS pathway. Our results show that indeed the N-terminal domain and in particular the Glu 2 and Val 3 aa residues are involved in the inhibition of the RIG-I pathway, as individual mutations of these two aa impairs the ability of G protein to interfere with the RIG-I/MAVS interaction, using an overexpression system, and with the recruitment of RIG-I and ER-MAM to mitochondria/MAVS, resulting in enhanced activation of NF-κB and IRF-3, and their downstream target genes.

Although hMPV is a leading cause of both upper and lower respiratory tract infections in infants, elderly and immunocompromised patients worldwide, no treatment or vaccine is currently available [1]. A recombinant hMPV strain lacking G protein is under investigation at NIAID as a potential vaccine candidate. There is some evidence that hMPV G protein may not be very immunogenic and therefore dispensable for mounting an effective immune response [3], [4]. This is the rationale for considering a virus missing the G protein, which is attenuated *in vivo*, as a potential vaccine candidate. On the other hand, in a rodent model of infection rhMPV-ΔG induces two and a half log2 lower neutralizing antibody titers compared to infection with rhMPV-WT. Furthermore, there was also detectable viral replication in the upper airways of animals immunized with rhMPV-ΔG, but not with rhMPV-WT, following viral challenge with naïve hMPV, suggesting that G protein may play an important role in inducing protective immune responses [3], [4]. Indeed, glycoprotein G from other viruses has been shown to be important for eliciting protective immune responses. For examples, the glycoprotein of RSV and Epstein-Barr virus has been shown to be important for inducing protective immunity against viral reinfection [39]–[41]. The data from our current and previous studies clearly show that G is an important regulator for host innate immunity, both in airway epithelial cells and immune cells [18], [19], suggesting that hMPV G could also modulate protective immunity, as innate immune responses have consequential influence on adaptive immune response. Therefore, the findings presented in our paper can have an important translational application when used to improve the design of the current vaccine candidates. For example, generation of a mutant virus which has no or reduced inhibitory activity and contains the fewest changes, optimally as single amino acid modifications, retaining partial or full G protein expression, could be important for generation of fully protective immune responses. We are currently investigating the effect of hMPV G protein expression in hMPV-induced innate and adaptive immune responses *in vivo*, using a mouse model of infection [42]–[44], by comparing the type and magnitude of these responses following infection with rhMPV-WT versus the -ΔG and rhMPV carrying individual G protein site mutations.

## Supporting Information

Figure S1 **Effect of G protein deletion on innate immune response in U4A cells.** U4A cells were infected with rhMPV-WT or rhMPV-ΔG, and harvested at 6, 15 and 24 h p.i. to measure secretion of IL-8 (A) and RANTES (B) by ELISA. Cell lysates were subjected to Western blot to compare the viral protein expression in response to WT and ΔG infection (C). Data shown are representative of two independent experiments. **, *P*<0.01 relative to rhMPV-WT.
(TIFF)

Figure S2 **Expression levels of hMPV G protein full length, extracellular domain or mutants.** A549 cells were transfected with a plasmid containing either full length G or G extracellular domain (Gc) (**A**) or G mutants carrying Ala mutation of individual amino acids, as indicated (**B**), or the corresponding empty vector. Cells were harvested 30 h post-transfection to prepare total cell lysates, after collection of cell supernatants. Expression of full length G and Ala site mutants was detected in total cell lysates, while hMPV cG expression was detected in cell supernatants by Western blot assays using an anti-V5 antibody. Membranes were stripped and reprobed with anti-β-actin, as control for comparable loading of samples. NS indicates a non-specific band. Data shown are representative of two independent experiments.
(TIFF)

Figure S3 **Amino acid residues Glu 2 and Val 3 of hMPV G are responsible for inhibiting the interaction between RIG-I and MAVS.** 293 cells were transfected with a fixed amount of plasmids encoding Flag-tagged RIG-I and EGFP-tagged MAVS, and a plasmid expressing either V5-tagged G, E2 G mutant, V3 G mutants, N or their control vector (CV). Total cell lysates were immunoprecipitated with anti-Flag antibody to pull down RIG-I, followed by Western blot using anti-EGFP antibody to detect associated MAVS. Total cell lysates were subjected to Western blot to determine levels of hMPV G and site mutants, RIG-I-N and MAVS expression. Data are representative of two independent experiments.
(TIFF)
