# Supplementary figures and images for: Human Metapneumovirus Glycoprotein G Disrupts Mitochondrial Signaling in Airway Epithelial Cells
Source: PLoS One. 2013 Apr 23;8(4):e62568. doi: 10.1371/journal.pone.0062568 (PMC3633857; doi:10.1371/journal.pone.0062568)

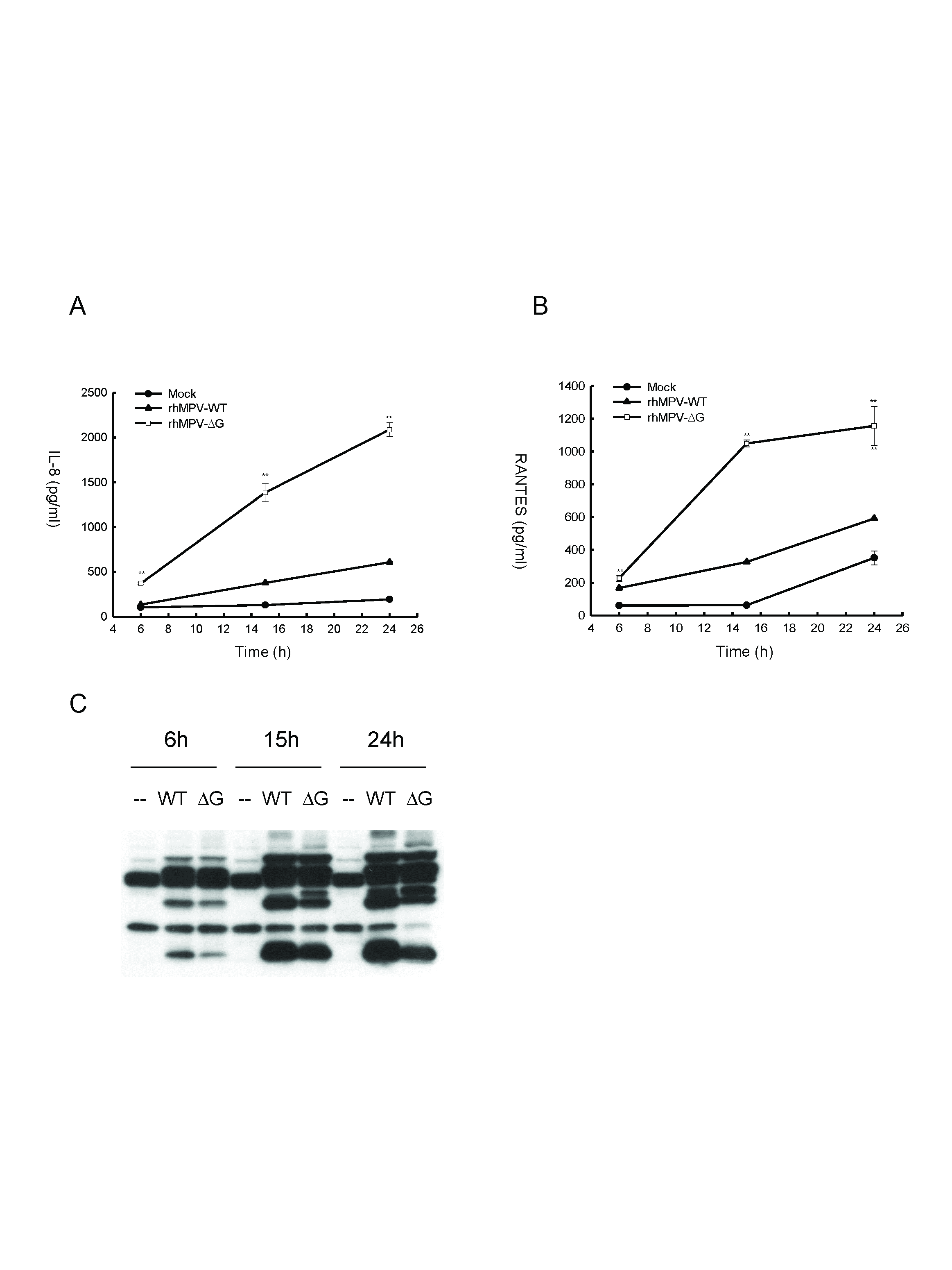

Supplement: Figure S1 — Effect of G protein deletion on innate immune response in U4A cells. U4A cells were infected with rhMPV-WT or rhMPV-ΔG, and harvested at 6, 15 and 24 h p.i. to measure secretion of IL-8 (A) and RANTES (B) by ELISA. Cell lysates were subjected to Western blot to compare the viral protein expression in response to WT and ΔG infection (C). Data shown are representative of two independent experiments. **, P<0.01 relative to rhMPV-WT. (TIFF) [file pone.0062568.s001.tiff]

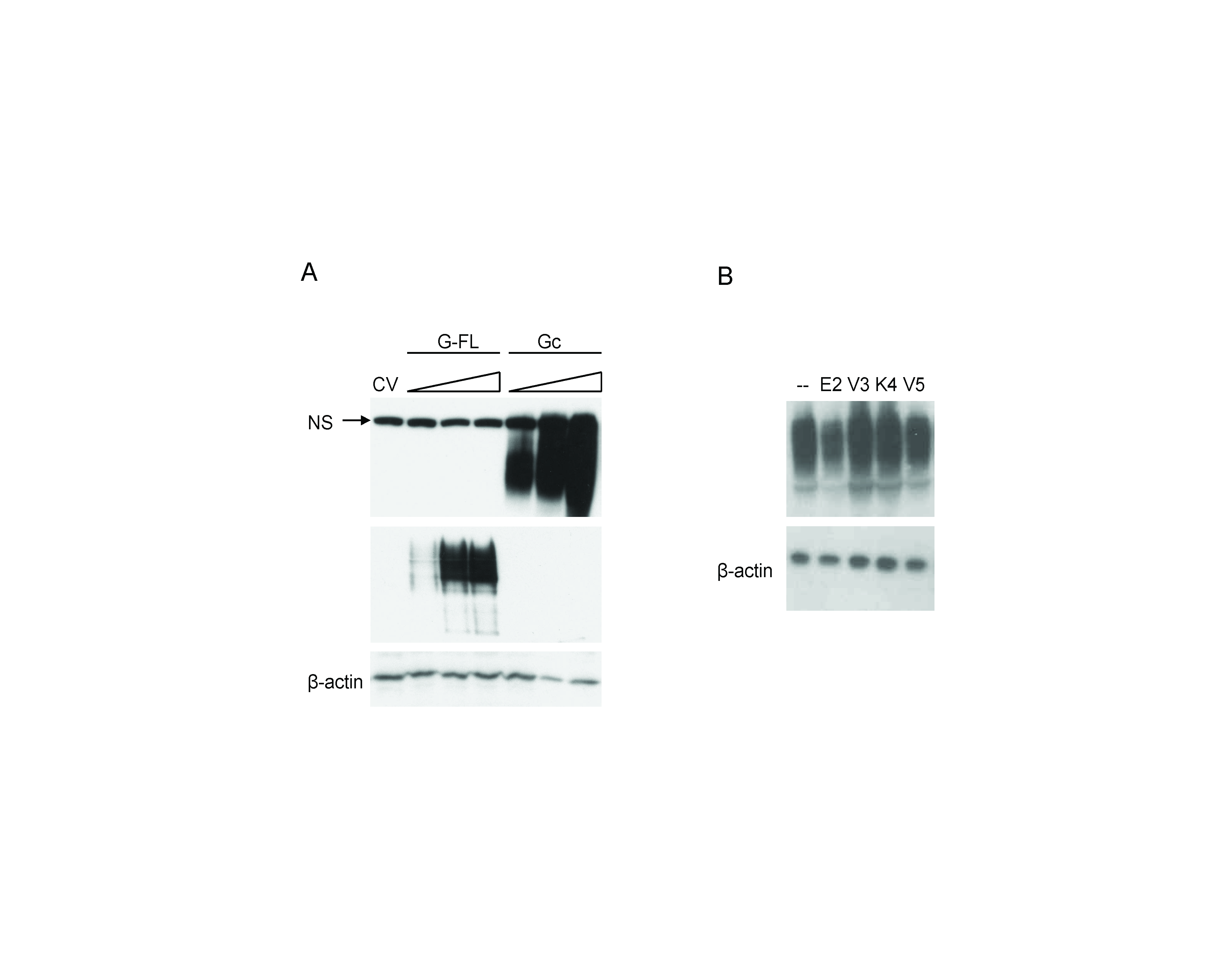

Supplement: Figure S2 — Expression levels of hMPV G protein full length, extracellular domain or mutants. A549 cells were transfected with a plasmid containing either full length G or G extracellular domain (Gc) (A) or G mutants carrying Ala mutation of individual amino acids, as indicated (B), or the corresponding empty vector. Cells were harvested 30 h post-transfection to prepare total cell lysates, after collection of cell supernatants. Expression of full length G and Ala site mutants was detected in total cell lysates, while hMPV cG expression was detected in cell supernatants by Western blot assays using an anti-V5 antibody. Membranes were stripped and reprobed with anti-β-actin, as control for comparable loading of samples. NS indicates a non-specific band. Data shown are representative of two independent experiments. (TIFF) [file pone.0062568.s002.tiff]

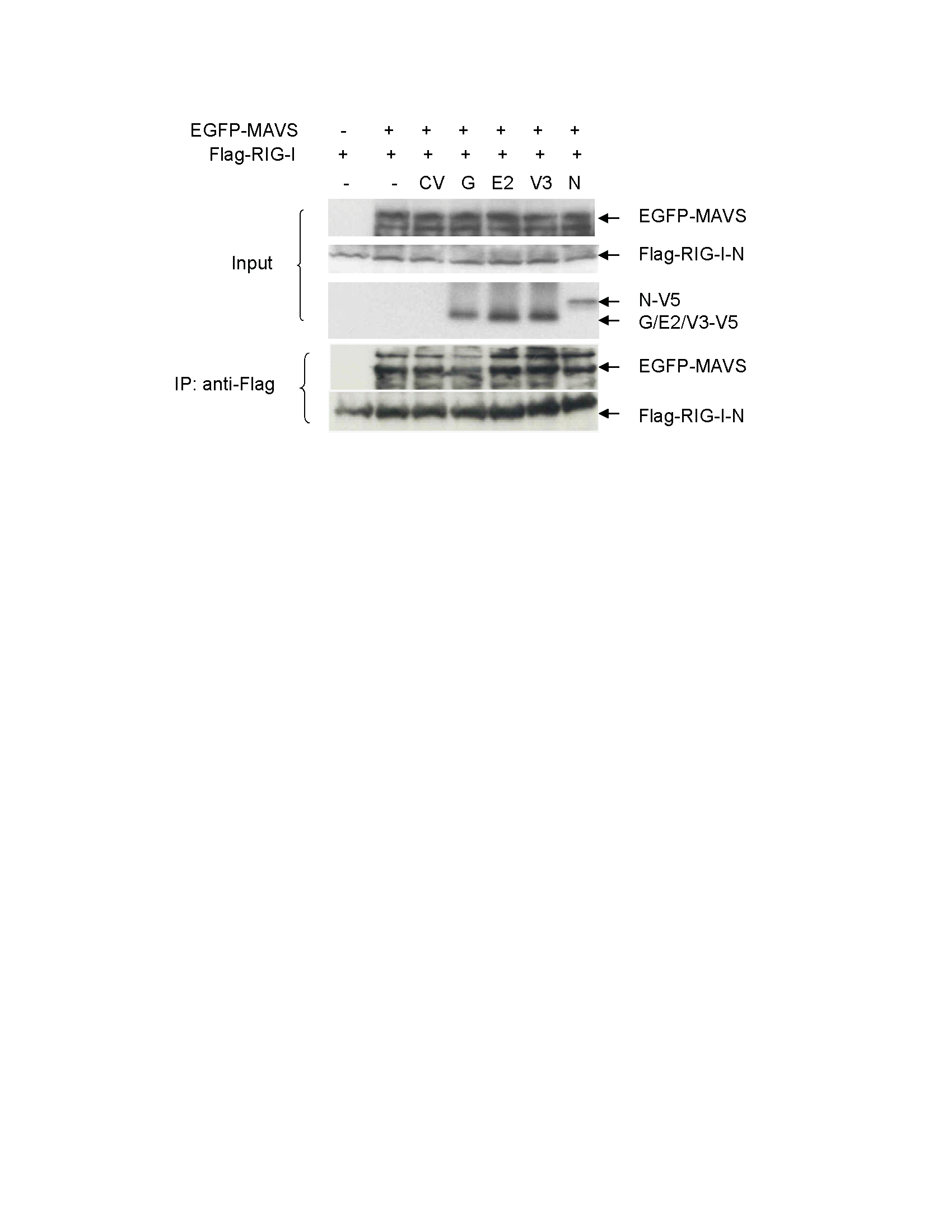

Supplement: Figure S3 — Amino acid residues Glu 2 and Val 3 of hMPV G are responsible for inhibiting the interaction between RIG-I and MAVS. 293 cells were transfected with a fixed amount of plasmids encoding Flag-tagged RIG-I and EGFP-tagged MAVS, and a plasmid expressing either V5-tagged G, E2 G mutant, V3 G mutants, N or their control vector (CV). Total cell lysates were immunoprecipitated with anti-Flag antibody to pull down RIG-I, followed by Western blot using anti-EGFP antibody to detect associated MAVS. Total cell lysates were subjected to Western blot to determine levels of hMPV G and site mutants, RIG-I-N and MAVS expression. Data are representative of two independent experiments. (TIFF) [file pone.0062568.s003.tiff]
